# Risk markers for suicidality in autistic adults

**DOI:** 10.1186/s13229-018-0226-4

**Published:** 2018-07-31

**Authors:** Sarah Cassidy, Louise Bradley, Rebecca Shaw, Simon Baron-Cohen

**Affiliations:** 1 School of Psychology, University of Nottingham, University Park, Nottingham, NG7 2RD UK; 2 Centre for Innovative Research across the Life Course, Coventry University, Coventry, UK; 3 Autism Research Centre, University of Cambridge, Cambridge, UK; 4 Coventry and Warwickshire Partnership Trust, Coventry, UK; 5 Cambridge Lifetime Asperger Syndrome Service (CLASS), Cambridgeshire and Peterborough NHS Foundation Trust, Cambridge, UK

**Keywords:** Autism spectrum condition, Autistic traits, Suicidality, Non-suicidal self-injury, NSSI, SBQ-R, NSSI-AT, Risk markers, Mental health, Depression, Anxiety

## Abstract

**Background:**

Research has shown high rates of suicidality in autism spectrum conditions (ASC), but there is lack of research into *why* this is the case. Many common experiences of autistic adults, such as depression or unemployment, overlap with known risk markers for suicide in the general population. However, it is unknown whether there are risk markers unique to ASC that require new tailored suicide prevention strategies.

**Methods:**

Through consultation with a steering group of autistic adults, a survey was developed aiming to identify unique risk markers for suicidality in this group. The survey measured suicidality (SBQ-R), non-suicidal self-injury (NSSI-AT), mental health problems, unmet support needs, employment, satisfaction with living arrangements, self-reported autistic traits (AQ), delay in ASC diagnosis, and ‘camouflaging’ ASC. One hundred sixty-four autistic adults (65 male, 99 female) and 169 general population adults (54 males, 115 females) completed the survey online.

**Results:**

A majority of autistic adults (72%) scored above the recommended psychiatric cut-off for suicide risk on the SBQ-R; significantly higher than general population (GP) adults (33%). After statistically controlling for a range of demographics and diagnoses, ASC diagnosis and self-reported autistic traits in the general population significantly predicted suicidality. In autistic adults, non-suicidal self-injury, camouflaging, and number of unmet support needs significantly predicted suicidality.

**Conclusions:**

Results confirm previously reported high rates of suicidality in ASC, and demonstrate that ASC diagnosis, and self-reported autistic traits in the general population are independent risk markers for suicidality. This suggests there are unique factors associated with autism and autistic traits that increase risk of suicidality. Camouflaging and unmet support needs appear to be risk markers for suicidality unique to ASC. Non-suicidal self-injury, employment, and mental health problems appear to be risk markers shared with the general population that are significantly more prevalent in the autistic community. Implications for understanding and prevention of suicide in ASC are discussed.

## Background

There are elevated rates of suicidality in adults diagnosed with autism spectrum conditions (ASC) [1–5]. However, suicidality in ASC is poorly understood, and there is a paucity of research exploring *why* adults with ASC (henceforth, autistic adults) may be at increased risk [6]. Although a number of studies have explored suicidality in autistic adults, no study has yet utilised a suicidality assessment tool with evidence of validity [4, 7, 8]. Non-suicidal self-injury (NSSI) is a risk factor for suicide attempts in the general population [9]. However, to our knowledge, only one study has ever explored NSSI in a small sample of autistic adults using a validated instrument but did not explore associations with suicidality [10]. Clearly, it is crucial to better understand suicidality in autistic adults, and associated risk markers, using instruments with evidence of validity (albeit not yet in autistic adults). Given the paucity of literature in the area of suicide in ASC research, it is important to engage with the autistic community in the refinement of research priorities to speed up progress and benefit the end users of research [11]. This is the aim of the current study.

Suicidal thoughts and behaviours are significantly increased in autistic adults compared to the general population and other clinical groups. In a large sample of 374 adults newly diagnosed with Asperger syndrome (AS; autism without language delay or intellectual disability), 66% had contemplated suicide, significantly higher than the general population (17%) and patients with psychosis (59%); 35% had planned or attempted suicide [2], higher than previous estimates of attempted suicide in general and university populations (2.5–10%) [12–14]. Only one study has ever explored whether autistic people are more at risk of dying by suicide than the general population; this population study in Sweden showed that autistic people were significantly more likely to die by suicide (0.31%) compared to the general population (0.04%) [15].

Traits characteristic of autism are also significantly associated with suicidality in those with [2], and without ASC diagnosis [16–18]. ASC diagnosis has also recently been found to be an independent risk marker for suicide attempts independent of demographic characteristics and co-occurring diagnoses [19]. These findings suggest that ASC explains additional variance in suicidality, not accounted for by other well-known risk markers in the general population which are more prevalent in ASC, such as depression [20–22] or social isolation [23, 24], which have been associated with increased risk of suicidality in ASC [2, 15, 17, 25, 26]. Hence, there may be as yet unknown unique risk markers for suicidality in ASC that are not shared with the general population or other clinical groups, requiring adapted suicide prevention strategies [6].

Studies exploring the characteristics of suicidality in ASC could provide important clues for possible unique risk markers in this group. For example, the highest rates of suicidal ideation (66%) were reported in adults newly diagnosed with AS, who had struggled without support [2]. Age of diagnosis and adequate access to post-diagnostic support could therefore be particularly important in preventing suicidality in ASC [2]. However, many children and adults diagnosed with ASC not only struggle to obtain their diagnosis, but also struggle to obtain post-diagnostic support [27–29]. Lack of tangible social support has been associated with increased risk of suicidality, indirectly through depression [25].

In the general population, the global male to female ratio of deaths by suicide is estimated to be 1.7 [30], indicating that males are more likely to die by suicide than females. However, in the one available study exploring death by suicide in the autistic community, autistic females without intellectual disability (ID) were more at risk of dying by suicide (0.32%) compared to autistic males (0.3%); opposite to the general population where males (0.05%) were more likely to die by suicide than females (0.03%) [15]. Autistic females have been under-researched, and it has been recognised that this group may also be under-diagnosed [29, 31, 32]. Autistic people report attempting to camouflage their ASC in order to try and fit in in social situations, which may delay obtaining a timely ASC diagnosis and negatively affect their mental health [31–33]. However, no study has quantitatively measured associations between ‘camouflaging’ and risk of mental health difficulties or suicidality in both autistic males and females.

In addition to lack of research into possible autism specific risk markers for suicidality, some potentially common risk factors for suicidality in those with and without ASC diagnosis have very different conceptualisations that have resulted in them being overlooked by researchers and clinicians. For example, self-injurious behaviour in ASC [34] is conceptualised rather differently than NSSI in the general population, as primarily a restricted and repetitive behaviour characteristic of ASC [35]. By contrast NSSI in the general population is considered a possible risk marker for later suicide attempts [9]. Only one study has explored NSSI in autistic adults without co-occurring ID using a tool validated for online research in non-clinical populations [10] (non-suicidal self-injury assessment tool (NSSI-AT)) [36]. The rate of NSSI in ASC was elevated (50%) compared to college students (17%) and adult community samples (23%), but the phenomenology of NSSI was broadly similar between those with and without ASC [10]. Importantly, this suggests that NSSI could be more prevalent in ASC than that in the general population, and could potentially be a previously unexplored common risk factor for suicidality in ASC and the general population.

Previous research has taken a piecemeal approach to furthering our understanding of suicidality in ASC. Important limitations include the fact that no suicidality studies in ASC have used a suicidality assessment tool with evidence of validity in this group [7, 8], and very few studies have included a comparison group [3]. Studies have also failed to disentangle common shared and unique risk markers for suicidality in autistic and general populations, which is key to understanding and preventing suicide in ASC [6].

The current study thus aimed to address these pitfalls in previous suicidality in ASC research. First, we used both a review of the available literature, and consultation with a steering group of autistic adults who have experienced suicidality, to ensure that we identified a range of high priority risk markers for suicidality in autism, some of which may be unique to this group. Second, we are the first to utilise a well-validated suicidality assessment tool (the Suicide Behaviours Questionnaire-Revised (SBQ-R)) [37] in autistic adults (confirmed in a systematic review) [7], and NSSI assessment tool previously utilised in autistic adults (NSSI-AT) [10]. We also include a general population comparison group. Hence, we are able to explore whether autistic adults are at increased risk of suicidality compared to the general population, while controlling for known common risk factors for suicidality (e.g. age, sex, mental health problems, employment, living situation). We also explore for the first time a potentially unique risk marker for suicidality and NSSI in ASC males and ASC females—camouflaging ASC in order to cope in social situations—as well as age of ASC diagnosis, and unmet support needs. We also explore whether NSSI is an independent risk marker for suicidality in those with and without ASC, and whether autistic traits are an independent risk marker for suicidality in the general population without ASC diagnosis.

## Method

### Participants

The ASC group comprised 164 adults (65 males; 99 females) who self-reported a diagnosis of ASC from a trained clinician, and a majority (81.1%) confirmed the clinic where this diagnosis was obtained. The general population group comprised 169 adults (54 males; 115 females). Participants were aged between 20 and 60 years old (Table 1). There were no significant differences in age (*t*(331) = .657, *p* = .511) or sex ratio (*χ*^*2*^ (1) = 2.14, *p* = .14) between the ASC and general population group. The ASC group scored significantly higher on the Autism-Spectrum Quotient (AQ) (36.42) than the general population group (19.87) (*t*(331) = .657, *p* < .001). See Table 1 for group demographics.

**Table 1:** Participant characteristics

Variables	Group			
	GP male (*n* = 54)	GP female (*n* = 115)	ASC male (*n* = 65)	ASC female (*n* = 99)
	Mean (SD)/*n* (%)			
Age	39.11 (10.09)	41.48 (11.18)	41.52 (11.73)	38.89 (10.47)
AQ total score	22.96 (8.56)	18.43 (7.12)	35.38 (7.5)	37.1(8.33)
Age diagnosed with ASC	–	–	34.55 (14.75)	35.06 (11.83)
% Lifetime ‘camouflage’	–	–	58 (89.2)	90 (90.9)
Camouflage total score	–	–	12.9 (4.06)	14.7 (3.61)
% Non-suicidal self-injury	18 (33.3)	32 (28.1)	35 (53.8)	71 (74)
Suicidality				
SBQ-R total score	7.48 (3.7)	6.36 (3.08)	10.14 (3.99)	10.56 (3.98)
% ≥ general population cut off	27 (50)	49 (42.6)	52 (80)	79 (79.8)
% ≥ psychiatric population cut-off	22 (40.7)	35 (30.4)	45 (69.2)	73 (73.7)
% Lifetime suicide attempt	7 (13)	7 (6.1)	21 (32.3)	42 (42.4)
ASC subtype				
HFA/AS	–	–	51 (78.5)	85 (85.9)
Autism/classic autism	–	–	0 0)	2 (2)
ASC	–	–	7 (10.8)	7 (6.9)
PDD/PDD-NOS	–	–	1 (1.5)	1 (1)
Other	–	–	6 (9.2)	4 (4)
Education type				
Mainstream	53 (98.1)	113 (98.3)	59 (98.1)	88 (88.9)
Home	1 (1.9)	2 (1.7)	1 (1.5)	2 (2)
Special	0 (0)	0 (0)	3 (4.6)	4 (4)
Private/boarding	0 (0)	0 (0)	2 (3.1)	5 (5.1)
Support				
Need/receive support	16 (29.6)	36 (31.3)	51 (78.5)	75 (76.5)
Unmet support needs*	2.12 (1.78)	1.3 (1.47)	3.1 (2.44)	3.43 (2.25)
Treatment				
Current treatment (total)	28 (51.9)	60 (53.1)	51 (78.5)	77 (77.8)
For mental health	27 (93.1)	51 (76.1)	44 (77.2)	71 (76.3)
For suicidal thoughts	9 (31)	8 (11.9)	14 (24.6)	25 (26.9)
For self-injury	3 (10.3)	2 (3)	4 (7)	9 (9.7)
Other	2 (6.9)	6 (9)	8 (14)	14 (14)
Living arrangements				
Living independently	15 (27.8)	26 (22.6)	18 (27.7)	30 (30.3)
Living with parents	5 (9.3)	5 (4.3)	15 (23.1)	15 (15.2)
Living with flatmate(s)	4 (7.4)	8 (7)	2 (3.1)	3 (3)
Live with friend(s)	0 (0)	3 (2.6)	1 (1.5)	0 (0)
Living with a partner and/or dependent(s)	29 (53.7)	71 (61.7)	21 (32.3)	44 (44.4)
Living in supported accommodation	0 (0)	0 (0)	2 (3.1)	1 (1)
Living with a carer	0 (0)	0 (0)	1 (1.5)	1 (1)
Other	1 (1.9)	2 (1.7)	5 (7.7)	5 (5.1)
Occupational status				
Employed	41 (75.9)	94 (81.7)	30 (46.2)	51 (51.5)
Volunteering	2 (3.7)	6 (5.2)	3 (4.6)	9 (9.1)
Student	5 (9.3)	6 (5.2)	6 (9.2)	15 (15.2)
Unemployed/unable to work	4 (7.4)	9 (7.8)	25 (38.5)	22 (22.2)
Retired	2 (33.3)	0 (0)	1 (1.5)	2 (2)
Mental health or other condition				
≥1 mental health or other condition	29 (53.7)	66 (57.4)	51 (78.5)	92 (92.9)
Current medication for mental health condition	10 (34.5)	26 (39.4)	26 (51)	56 (60.9)
Depression	25 (46.3)	51 (44.3)	47 (72.3)	84 (84.8)
Anxiety	19 (35.2)	42 (36.5)	40 (61.5)	77 (77.8)
Obsessive compulsive disorder	0 (0)	3 (2.6)	7 (10.8)	17 (17.2)
Bipolar disorder	1 (1.9)	2 (1.7)	2 (1.7)	6 (3.7)
Personality disorder	1 (1.9)	4 (3.5)	5 (7.7)	18 (18.2)
Schizophrenia	0 (0)	0 (0)	2 (3.1)	4 (4)
Anorexia nervosa	0 (0)	4 (3.5)	1 (1.5)	8 (8.1)
Bulimia	0 (0)	1 (0.9)	0 (0)	2 (2)
Myalgic encephalopathy	0 (0)	3 (2.6)	3 (4.6)	10 (10.1)
Tourettes	0 (0)	0 (0)	2 (3.1)	2 (2)
Epilepsy	1 (1.9)	4 (3.5)	1 (1.5)	4 (4)
Other	4 (7.4)	4 (3.5)	10 (15.4)	21 (21.2)
Developmental condition				
≥1 developmental condition	2 (3.7)	1 (0.9)	15 (23.1)	22 (22.2)
Dyspraxia	1 (1.9)	1 (0.9)	7 (3.9)	11 (11.1)
Learning disability	1 (0)	0 (0)	1 (1.5)	0 (0)
Learning difficulty	0 (0)	0 (0)	0 (0)	2 (2)
Dyscalculia	0 (0)	0 (0)	2 (31)	1 (1)
Dyslexia	2 (3.7)	0 (0)	5 (7.7)	8 (8.1)
Attention deficit hyperactivity disorder	0 (0)	0 (0)	2 (3.1)	9 (9.1)
Developmental delay	0 (0)	0 (0)	0 (0)	1 (1)
Other	0 (0)	1 (0.9)	2 (3.1)	4 (4)

*NB, unmet support needs calculated by (total *n* areas support ideally liked—total *n* areas support actually received)

Participants were recruited from research volunteers databases located in the Autism Research Centre at the University of Cambridge. Autistic adults and their family members across the UK and internationally register in the Cambridge Autism Research Database (CARD) (https://www.autismresearchcentre.net/). General population adults without an autism diagnosis or autistic family members register at a separate website (https://www.cambridgepsychology.com/login). Volunteers register in these databases to receive information about a variety of psychology research projects and not mental health specifically. Additionally, participants were recruited from online adverts.

### Measures

#### Survey development

An online questionnaire exploring mental health, self-injury, and thoughts of ending life was developed for the current study in partnership with a steering group of eight adults diagnosed with ASC (6 females, 2 males) through a series of 6 focus groups. Given the topic of the survey, all steering group members were recruited by advertising for autistic adults who would like to share their experience to influence research and improve support for mental health problems, self-injury, and suicidality. The first three focus groups developed the topics to be captured in the questionnaire. First, the researchers proposed a number of topics thought to be important contributors to mental health and suicidality in autism, and the focus group fed back on the relevance and importance of these proposed topics, and whether any important topics were missing. This ensured that a large array of possible risk markers was prioritised for the study. Subsequent focus groups discussed participants’ experiences of the topics. The researchers then developed a survey to capture these topics and experiences. The steering group provided feedback on three drafts of the survey to ensure that the questions were comprehensive, relevant, and clear.

#### Demographics

Participants who completed the online survey provided information on age, biological birth sex, education, employment, living situation, diagnosed developmental and mental health conditions, current medication, whether they were currently receiving any treatment for mental health problems, suicidal thoughts, self-injury or other reason. Participants also reported whether they need or currently receive support, and if yes, were asked (a) in which areas they would ideally like support in (in the home, with employment, health care, mental health care, finance, social activities, in the community, organisation, mentoring, education, other); and (b) in which of these areas they actually receive support. Unmet support needs were thus calculated as the mismatch between the number of areas participants actually received support, compared to the number of areas participants would ideally like support (unmet support needs = *n* areas support ideally liked—*n* areas support actually received) (Table 1).

#### Camouflaging

A brief set of four questions were designed to quantify tendency to camouflage in the current study. Autistic adults were asked “Have you ever tried to camouflage or mask your characteristics of ASC to cope with social situations? For example, have you ever tried to copy or mimic other people’s behaviour to try and fit in (e.g. copying another person’s accent or mannerisms), or tried to mask or hide your symptoms of ASC from other people?” If participants responded yes, they subsequently (a) specified the areas in which they camouflage (work, educational settings, social gatherings, when visiting the doctors, when visiting a health professional, at home, with friends, other); (b) the overall frequency they camouflage on a scale from 1 (never) to 6 (always (over 90% of social situations)); and (c) overall amount of the day they spend camouflaging on a scale from 1 (none of my waking time) to 6 (all of my waking time (over 90% of social situations)). Scores were calculated as the sum of number of areas (maximum 8), overall frequency (maximum 6), and overall amount (maximum 6), with a maximum score of 20 overall. Internal consistency for the whole scale was acceptable in the ASC group (α = .75).

#### Autism-Spectrum Quotient (AQ)

The Autism-Spectrum Quotient (AQ) is a 50-item questionnaire assessing the number of self-reported autistic traits [38]. The AQ has been shown to reliably distinguishing those with and without a diagnosis of ASC [38, 39], with scores ≥ 26 indicating potential diagnosis of ASC [40].

#### Non-suicidal self-injury (NSSI)

The non-suicidal self-injury assessment tool (NSSI-AT) [36] was used to screen for presence of any form of lifetime NSSI in the current sample. Participants were first asked the screening question “Have you ever hurt your body (e.g. cut, carve, burn, scratch really hard, punch) on purpose but without wanting to end your life?” If yes, participants then completed sections A–B of the NSSI-AT to confirm that suicidality was not the primary reason for their self-harm. Subsequently, responses were classified as endorsing lifetime NSSI, or no lifetime NSSI.

The NSSI-AT was developed as a research tool to assess NSSI online in non-clinical populations and has previously been shown to have adequate measurement properties in college students; test-retest reliability for any form of NSSI was 0.74, with moderate correlations with related behavioural problems [36]. One study has previously used the NSSI-AT in an ASC adult sample and found evidence in support of similar phenomenology of NSSI in those with and without ASC [10].

#### Suicidality

Participants completed the Suicide Behaviours Questionnaire-Revised (SBQ-R) [37], a 4-item self-report questionnaire that assesses lifetime suicidal behaviour, suicide ideation over the past 12 months, threat of suicide attempt, and likelihood of suicidal behaviour in the future. The SBQ-R has been validated for use in general population and clinical samples to reliably distinguish suicide attempters from non-attempters [37], and is widely used in research, with moderate-strong evidence in support of internal consistency, structural validity, hypothesis testing, and criterion validity in clinical and non-clinical samples [7]. Internal consistency for the whole scale was acceptable in both autistic adults (α = .76) and general population adults (α = .768).

#### Ethical approval

The current study received ethical approval from Coventry University Psychology Ethics Committee and was approved by the autism steering group who fed back on the questionnaire, and the scientific advisory group at the Autism Research Centre, University of Cambridge, prior to recruiting participants registered in the Cambridge Autism Research Database (CARD).

#### Procedure

Participants with and without ASC diagnosis were invited to complete an online survey about understanding and preventing mental health problems, self-injury, and suicidality. Participants could take part regardless of prior experience of mental health difficulties, self-injury or suicidality. Participants read the participant information and indicated informed consent to participate via an online form. Participants were fully briefed about the nature of the research, that they could skip sections and/or questions that made them feel uncomfortable, and were provided information about relevant support services before and after taking part in the study. Participants subsequently completed questions on demographics, diagnoses (mental health, developmental conditions, and ASC), NSSI-AT, camouflaging, AQ, SBQ-R, current treatment (for mental health, self-injury, or suicidality), and support (areas in which support was actually received and ideally liked but not yet received).

#### Analysis approach

Data were analysed using SPSS 24. Chi-square analysis was used to explore group differences in frequency of lifetime NSSI, lifetime experience of camouflaging, and demographics, with odds ratios (with 95% confidence intervals) calculated as a measure of effect size. Independent samples *t* tests were used to compare total scores on the SBQ-R, AQ, and camouflaging questionnaires between groups, with Cohen’s *d* as a measure of effect size (where 0.2 = small, 0.5 = medium, and 0.8 = large effect) [41]. One sample *t* tests compared SBQ-R total scores to established cut-offs in general and psychiatric populations. Spearman’s correlations were used to explore inter-correlations between all variables in each group (where 0.1 = small, 0.3 = medium, and 0.5 = large effect). Multiple hierarchical regressions subsequently explored whether significant associations between demographics and diagnoses with suicidality remained when controlling for significant covariates.

The SBQ-R was non-normally distributed. Analyses were therefore undertaken using bootstrapping techniques, a robust analysis technique which is reliable even when assumptions of a symmetric distribution are not met [42]. Utilising this robust analysis technique did not alter the pattern of results, with similar direction and magnitude of effects and statistical significance found using bootstrapping or normal analytic approach; therefore, untransformed results are reported for ease of interpretation.

## Results

### Group comparisons

#### Suicidality

There was no significant difference in total SBQ-R scores between autistic males and autistic females (*t*(162) = .671, *p* = .503), so results were pooled. A one sample *t* test showed that autistic adults SBQ-R total scores were significantly higher than the recommended cut-off for the general population (7) (*t*(163) = 10.92, *p* < .001), and psychiatric populations (8) (*t*(163) = 7.71, *p* < .001) [33]. A majority (72%) of autistic adults scored at or above the cut-off for psychiatric populations (8) (Table 1).

There was a significant difference in total SBQ-R scores between general population (GP) males and females (*t*(167) = 2.06, *p* = .041), so data from males and females were analysed separately. One sample *t* tests showed that GP males SBQ-R scores were not significantly different from the recommended cut-off for the general (*t*(53) = .956, *p* = .343) or psychiatric population (*t*(162) = .671, *p* = .503). GP females scored significantly lower than the recommended cut off for the general (*t*(114) = 2.211, *p* = .029) and psychiatric population (*t*(114) = 5.694, *p* < .001) (Table 1).

Autistic adults scored significantly higher on the SBQ-R than GP adults (*t*(331) = 9.131, *p* < .001, *d* = 1) and were significantly more likely to score above the psychiatric cut-off for suicide risk (72%) than GP adults (33.7%) (*χ*^*2*^(1) = 48.77, *p* < .001, OR 5.04, 95% CI 3.16–8.04) (Table 1).

#### NSSI

Significantly more autistic females (74%) reported NSSI than autistic males (53.8%) (*χ*^*2*^(1) = 6.97, *p* < .01, OR 2.43, 95% CI 1.25–4.74). There was no significant sex difference in NSSI in the GP group (*χ*^*2*^(1) = .486, *p* = .486). Autistic adults were significantly more likely to report lifetime NSSI (65%) than GP adults (29.8%) (*χ*^*2*^(1) = 42.91, *p* < .001, OR 4.55, 95% CI 2.86–7.23) (Table 1).

#### Demographics

Compared to the general population, autistic adults reported significantly lower satisfaction with their living arrangements (*t*(146) = 2.82, *p* = .005; *d* = .4) were significantly more likely to be unemployed (*χ*^*2*^(1) = 33.95, *p* < .001, OR 4.07, 95% CI 2.5–6.61), be diagnosed with at least one co-occurring developmental condition (*χ*^*2*^(1) = 34.02, *p* < .001, OR 16.12, 95% CI 4.86–53.47), at least one mental health or other condition (*χ*^*2*^(1) = 39.18, *p* < .001, OR 5.3, 95% CI 3.06–9.19), depression (*χ*^*2*^(1) = 43.1, *p* < .001, OR 4.86, 95% CI 2.98–7.91), anxiety (*χ*^*2*^(1) = 41.56, *p* < .001, OR 4.41, 95% CI 2.78–6.99), and report higher unmet support needs (*t*(176) = 4.91, *p* < .001; *d* = .87) (Table 1).

#### Camouflaging

There was no significant difference between autistic males (89.2%) and autistic females (90.9%) in terms of whether they attempted to camouflage their ASC in order to fit in in social situations (*χ*^*2*^(1) = .126, *p* = .723). However, autistic females scored significantly higher on the camouflaging questionnaire overall (14.7, *SD* 3.61) than autistic males (12.9, *SD* 4.06) (*t*(146) = 2.82, *p* = .005; *d* = .47) (Table 1).

### Predictors of suicidality in ASC

Table 2 shows the results of all inter correlations between variables in the ASC group. Lifetime NSSI, camouflaging, ADHD, depression, anxiety, unmet support needs, and satisfaction with living arrangements all significantly correlated with suicidality (total SBQ-R scores). However, age of diagnosis was not significantly correlated with any other variables.

**Table 2:** Means, standard deviations and inter-correlations for all variables in the ASC group

Variable	AQ	Age of ASC diagnosis	SBQ-R	NSSI	Lifetime camouflage	Camouflage score	Unmet support needs	≥ 1 developmental condition	ADHD	≥ 1 mental health/other condition	Depression	Anxiety	Satisfaction with living arrangements	Employed	Sex	Age at testing
AQ	–															
Age of ASC diagnosis	.147	–														
SBQ-R	.099	− .138	–													
NSSI	.126	− .125	.277*	–												
Lifetime camouflage	.053	− .048	.245*	.33*	–											
Camouflage score	.058	.107	.164*	.085	–	–										
Unmet support needs	.101	− .087	.247*	.109	.088	.085	–									
≥ 1 developmental condition	− .008	− .201*	.064	.009	.068	.073	−.023	–								
ADHD	− .02	− .113	.182*	.077	.088	.001	.058	.497*	–							
≥ 1 mental health/other condition	.259*	− .035	.365*	.188*	.182*	.112	.063	.032	.103	–						
Depression	.199*	.128	.322*	.088	.143	.076	− .035	−.093	−.048	.764*	–					
Anxiety	.161*	.012	.325*	.286*	.201*	.119	.079	.084	.062	.605	.59*	–				
Satisfaction with living arrangements	− .072	.081	− .257*	−.047	.003	.170*	− .386*	−.063	−.054	.034	.037	−.15	–			
Employed	− .174*	.023	− .114	.078	.037	.044	− .096	.021	.076	− .205*	− .143	− .102	.135	–		
Sex	.105	.105	.053	.208*	.028	.228*	.07	−.01	.118	.212*	.153	.176*	.212*	.052	–	
Age at testing	.104	.91*	− .137	− .152	− .073	.096	− .141	− .2*	− .134	− .017	.131	.028	.076	.03	− .117	–
Mean/%	36.42	34.85	10.4	64.63	90.2	13.99	3.29	22.5	6.7	87.19	79.88	71.34	68.47	28.66	39.63	39.93
SD	8.03	13.03	3.98	–	–	3.88	2.33	–	–	–	–	–	26.67	–	–	11.03

*Note: AQ*, Autism-Spectrum Quotient (total score); *SBQ-R*, Suicidal Behaviours Questionnaire-Revised (total score); *Lifetime camouflage*, attempting to camouflage autism in order to fit in in social situations; *Camouflage score*, total score on the camouflaging questionnaire; *Mismatch*, *(n* areas of support ideally liked – *n* areas actually received); *≥ 1* *developmental condition*, at least one co-occurring developmental condition; *≥ 1* *mental health/other condition*, at least one co-occurring mental health or other condition; *Sex*, % autistic male; *Age at testing*, age in years at testing. *Significant correlations *p* < .05

Hierarchical regression models were performed with total SBQ-R scores as the outcome variable. To statistically control for these variables, age at testing and gender were entered into the first step, and employment, satisfaction with living arrangements, developmental conditions, depression, and anxiety entered into the second step. The third step explored additional variance accounted for by the predictor variable. Separate models explored the additional predictive contribution of ASC diagnosis (in the combined ASC and GP groups), lifetime experience of NSSI, camouflaging questionnaire total scores, and unmet support needs (in the ASC sub-group), to the model. Age of ASC diagnosis was not explored further as a unique predictor given that this did not significantly correlate with any other variables (Table 2).

#### ASC diagnosis

In step one, the regression model containing sex and age significantly predicted SBQ-R scores (*F*(2,330) = 6.99, *p* < .001), accounting for 4.1% of the variance. In step two, employment, satisfaction with living arrangements, presence of at least one developmental condition, depression, and anxiety accounted for significantly more of the variance (33.4%) in SBQ-R scores (*F*(5,325) = 34.79, *p* < .001). In step three, autism diagnosis accounted for significantly more of the variance (4.5%) in SBQ-R scores (*F*(1,324) = 24.9, *p* < .001) (Table 3).

**Table 3:** Hierarchical regression with diagnostic group (ASC vs. general population) predicting SBQ-R

	B	SE B	β
Step 1			
Constant	12.408	1.135	
Sex	−.635	.460	− .074
Age	−.070	.020	− .188*
Step 2			
Constant	13.591	1.382	
Employed	−.768	.395	− .090
Satisfaction with living arrangements	−.045	.008	− .280*
≥1 developmental condition	.827	.567	.066
Depression	2.856	.482	.339*
Anxiety	.898	.474	.110
Step 3			
Constant	8.918	1.630	
Diagnostic group	2.038	.408	.249*

*Note: R*^*2*^ = .041 for step 1, *ΔR*^2^ = .334 for step 2, *ΔR*^*2*^ = .045 for step 3 (p < .001). *p < .001. *N =* 333

#### NSSI

In step one, the regression model containing sex and age did not significantly predict SBQ-R scores (*F*(2,158) = 1.99, *p* = .141), accounting for only 2.5% of the variance. In step two, employment, satisfaction with living arrangements, presence of at least one developmental condition, depression, and anxiety accounted for significantly more of the variance (19.9%) in SBQ-R scores (*F*(5,153) = 7.84, *p* < .001). In step three, NSSI accounted for significantly more of the variance (4%) in SBQ-R scores (*F*(1,152) = 6.78, *p* = .005) (Table 4).

**Table 4:** Hierarchical regressions with NSSI predicting SBQ-R in the ASC group

	B	SE B	β
Step 1			
Constant	12.283	1.661	
Sex	.153	.642	.019
Age at testing	− .055	.029	−.153
Step 2			
Constant	12.822	1.896	
Employed	− .261	.578	−.033
Satisfaction with living arrangements	− .037	.011	−.251*
≥ 1 developmental condition	− .194	.707	−.020
Depression	2.716	.903	.276*
Anxiety	.971	.809	.111
Step 3			
Constant	12.131	1.869	
NSSI	1.803	.631	.215*

*Note: R*^*2*^ = .012 for step 1, *ΔR*^*2*^ = .199 for step 2, *ΔR*^*2*^ = .04 for step 3 (p = .005). *p < .01*. N =* 161

#### Camouflaging

In step one, the regression model containing sex and age did not significantly predict SBQ-R scores (*F*(2,145) = .529, *p* = .59), accounting for only 0.7% of the variance. In step two, employment, satisfaction with living arrangements, at least one developmental condition, depression, and anxiety accounted for significantly more of the variance (20.7%) in SBQ-R scores (*F*(5,140) = 7.39, *p* < .001). In step three, camouflaging total scores accounted for significantly more of the variance (3.5%) in SBQ-R scores (*F*(1,139) = 6.56, *p* = .01) (Table 5).

**Table 5:** Hierarchical regression with camouflaging total scores predicting SBQ-R in the ASC group

	B	SE B	β
Step 1			
Constant	11.139	1.730	
Sex	.335	.668	.042
Age at testing	− .024	.030	− .068
Step 2			
Constant	12.033	2.043	
Employed	− .291	.599	− .037
Satisfaction with living arrangements	− .045	.012	− .307*
≥ 1 developmental condition	.275	.736	.029
Depression	2.725	.971	.270*
Anxiety	.803	.850	.090
Step 3			
Constant	10.217	2.126	
Camouflage score	.200	.078	.198*

*Note: R*^*2*^ = .006 for step 1, *ΔR*^*2*^ = .207 for step 2, *ΔR*^*2*^ = .035 for step 3 (p = .01). *p < .01*. N =* 148

#### Unmet support needs

In step one, the regression model containing sex and age did not significantly predict SBQ-R scores (*F*(2,123) = .233, *p* = .793), accounting for only 0.4% of the variance. In step two, employment, satisfaction with living arrangements, at least one developmental condition, depression, and anxiety accounted for significantly more of the variance (13.5%) in SBQ-R scores (*F*(5,118) = 3.7, *p* = .004). In step three, unmet support needs accounted for significantly more of the variance (3.1%) in SBQ-R scores (*F*(1,117) = 4.32, *p* = .04) (Table 6).

**Table 6:** Hierarchical regression with unmet support needs predicting SBQ-R in the ASC group

	*B*	*SE B*	β
Step 1			
Constant	11.738	1.759	
Sex	.142	.718	.018
Age at testing	− .020	.032	− .058
Step 2			
Constant	11.296	2.148	
Employed	.060	.690	.008
Satisfaction with living arrangements	− .028	.012	− .204*
≥ 1 developmental condition	.032	.830	.003
Depression	2.771	1.089	.264*
Anxiety	.826	.963	.090
Step 3			
Constant	8.394	2.537	
Unmet support needs	.329	.158	.195*

*Note: R*^*2*^ = .004 for step 1, *ΔR*^*2*^ = .135 for step 2, *ΔR*^*2*^ = .031 for step 3 (p = .04). *p < .05. *N =* 126

### Predictors of suicidality in the general population

Table 7 shows the results of all inter correlations between variables in the GP group. Self-reported autistic traits (AQ total scores), lifetime NSSI, depression, anxiety, satisfaction with living arrangements and employment all significantly correlated with suicidality (total SBQ-R scores).

**Table 7:** Means, standard deviations and inter-correlations for all variables in the general population group

Variable	AQ	SBQ-R	NSSI	Unmet support needs	≥ 1 developmental condition	≥ 1 mental health/other condition	Depression	Anxiety	Satisfaction with living arrangements	Employed	Sex	Age at testing
AQ	–											
SBQ-R	.329*	–										
NSSI	.009	.233*	–									
Unmet support needs	.277	.205	− .191	–								
≥ 1 developmental condition	.116	.097	.011	–	–							
≥ 1 mental health/other condition	.168*	.373*	.132	.052	.028	–						
Depression	.232*	.432*	193*	.091	−.059	.798*	–					
Anxiety	.206*	.301*	194*	−.136	.008	.663	.559*	–				
Satisfaction with living arrangements	.136	− .487*	− .119	−.193	−.005	.212*	− .181	− .173*	–			
Employed	.149	.185*	.029	.392	.044	.175*	.169*	− .115	.118	–		
Sex	− .269*	− .157*	−.054	−.238	.1	− .035	− .018	.013	.238*	.068	–	
Age at testing	− .15	− .257*	−.23*	−.121	.087	.082	− .032	− .148	.312*	.121	.102	–
Mean/%	19.86	6.72	29.8	1.56	2.9	56.2	44.97	19.24	78.44	7.69	31.95	42.72
*SD*	7.87	3.32	–	1.6	–	–	–	–	23.16	–	–	10.87

*Note: AQ,* Autism-Spectrum Quotient (total score); *SBQ-R,* Suicidal Behaviours Questionnaire-Revised (total score); *Mismatch, (n* areas of support ideally liked –*n* areas actually received); ≥ 1 developmental condition; *≥ 1 mental health*/*other condition*, at least one mental health or other condition; *Sex,* % male; *Age at testing,* age in years at testing. *Significant correlations *p <* .05

A hierarchical regression model was thus performed with total SBQ-R scores as the outcome variable. To statistically control for these variables, age at testing and gender were entered into the first step. To statistically control for additional co-variates, employment, satisfaction with living arrangements, developmental conditions, depression, and anxiety were entered into the second step. The third and final step explored much additional variance in suicidality was explained by self-reported autistic traits.

#### Autistic traits

In step one, the regression model containing sex and age significantly predicted SBQ-R scores (*F*(2,166) = 7.57, *p* < .001), accounting for 8.4% of the variance. In step two, employment, satisfaction with living arrangements, presence of at least one developmental condition, depression, and anxiety accounted for significantly more of the variance (31.5%) in SBQ-R scores (*F*(5,161) = 16.85, *p* < .001). In step three, self-reported autistic traits accounted for significantly more of the variance (3.2%) in SBQ-R scores (*F*(1,160) = 9.08, *p* = .003) (Table 8).

**Table 8:** Hierarchical regressions with autistic traits predicting SBQ-R in the general population group

Autistic traits	*B*	*SE B*	β
Step 1			
Constant	11.335	1.244	
Sex	− .940	.530	− .132
Age	−.074	.023	− .244*
Step 2			
Constant	5.690	3.193	
Employed	− .561	.517	− .068
Satisfaction with living Arrangements	− .051	.010	− .357*
≥ 1 developmental condition	3.410	1.554	.136*
Depression	2.321	.501	.349*
Anxiety	.119	.517	.017
Step 3			
Constant	2.597	3.281	
Autistic traits	.083	.027	.196*

*Note: R*^*2*^ = .084 for step 1, *ΔR*^*2*^ = .315 for step 2, *ΔR*^*2*^ = .032 for step 3 (*p =* .003). *p < .05*. N =* 169

## Discussion

Previous research exploring suicidality in ASC has failed to include adequately sized samples, matched comparison groups, explore risk or protective factors [2, 3, 6], or include validated suicidality assessment tools [7]. The current study aimed to address these weaknesses of previous research, to identify common and unique risk markers for suicidality in ASC. Specifically, whether there are unique aspects of ASC and autistic traits that increase risk of suicidality, after statistically controlling for common risk factors such as age, sex, employment, or mental health. We then explored possible unique risk factors which could explain increased risk of suicide in ASC, identified by our steering group of autistic adults: camouflaging one’s ASC in an attempt to fit in in social situations, age of ASC diagnosis, whether people felt they received the support they required, and NSSI. Previous studies have not systematically studied unique and common risk markers for suicidality in ASC compared to the general population, which has prevented development of tailored suicide prevention strategies for this group [6].

Results are consistent with previous findings that autistic adults are at significantly increased risk of suicidality compared to the general population [2]. A majority (72%) of autistic adults scored significantly above the recommended cut-off for suicide risk in psychiatric populations, significantly higher than general population adults (33%) with similar age and gender composition. This significant association between ASC diagnosis and suicidality remained when controlling for a number of demographics and diagnoses, known to increase or decrease risk of suicidality in the general population (employment, depression, anxiety, and satisfaction with living arrangements). Additionally, the significant association between self-reported autistic traits in the general population and suicidality remained after statistically controlling for these demographics and diagnoses. These results suggest that autism diagnosis and autistic traits explain significant additional variance in suicidality beyond a range of known risk factors, and are therefore independent risk markers for suicidality. This is consistent with research showing that ASC diagnosis is an independent risk marker for suicide attempts when controlling for a range of demographics and co-occurring diagnoses [19]. These findings suggest additional unique contributors to suicidality in ASC, which must be addressed in addition to important well-known factors such as mental health, employment, and living arrangements.

The current study explored a potentially unique risk marker for suicidality in ASC, identified by our steering group of autistic adults: tendency to camouflage one’s ASC in order to cope in social situations. Previous research [29, 32] and discussions with our steering group identified camouflaging as an important potential barrier to timely ASC diagnosis, and having a negative impact on mental health and risk of suicidality. Previous research has also suggested that camouflaging is primarily experienced by autistic females [31, 33], which may at least in part explain why this group has been under-diagnosed [43]. Results from the current study however showed subtle differences in camouflaging behaviour between autistic males and females: there was no sex difference in reporting whether one engages in camouflaging behaviour, but autistic females tended to report that they camouflaged across more situations, more frequently and more of the time than autistic males.

Camouflaging significantly predicted suicidality in the ASC group, after controlling for age, sex, presence of at least one developmental condition, depression, anxiety, employment, and satisfaction with living arrangements. Camouflaging and age of ASC diagnosis, and suicidality and age of ASC diagnosis were not significantly correlated. This suggests that camouflaging is directly associated with suicidality rather than in combination with delay in ASC diagnosis. Camouflaging also explained significant additional variance in suicidality above depression or anxiety, suggesting that the association with suicidality is, at least in part, independent of mental health. This is the first evidence of camouflaging being a unique independent risk factor for suicidality in ASC.

In order to engage in camouflaging, one must have insight into one’s own difficulties, how these may be negatively perceived by others, and have a strong motivation to adapt one’s social behaviour to be accepted. Understanding associations between these factors with camouflaging, and the consequent impact on mental health would be valuable. For example, autistic people who have greater insight into their own difficulties are more likely to be depressed than those with less insight [44], and autistic people are able to accurately predict how family members perceive them, despite being different to their own view [45]. It would be interesting to explore whether perspective taking ability and insight into one’s own difficulties increase likelihood of engaging in camouflaging behaviour with consequent negative impact on mental health and suicidality.

Importantly, our findings challenge the assumption that autistic people are socially unmotivated, consistent with calls for more accurate and useful autism research, embracing the unique nature of social interest in autism [46]. It is perhaps more accurate to acknowledge a “double empathy problem”, where autistic people are misinterpreted by non-autistic people and vice versa [45, 47, 48], which contribute to feelings of isolation among autistic people [49]. Increasing acceptance of autistic people in society could therefore lead to a reduced need for camouflaging and increased feelings of belonging—a protective factor for suicidality [17, 23].

Contrary to expectations, and discussions with our autistic steering group, age of ASC diagnosis was not significantly correlated with any other variables, such as mental health problems, suicidality, or NSSI. However, this may have been due to the fact that the mean age of ASC diagnosis was 34 years, and therefore, participants represent autistic people diagnosed in adulthood. Future research will need to explore whether those diagnosed in childhood are significantly less likely to experience mental health problems of suicidality compared to those diagnosed in adulthood. Another important theme identified from discussions with our steering group was lack of access to support, which could compound mental health difficulties and suicidality. Previous research has shown that the autistic community is disconnected from psychiatric services [18], as many practitioners are not trained in ASC [50]. The current study therefore quantitatively explored the mismatch between the number of areas an individual would ideally like support, compared to the number of areas they actually received support. These unmet support needs significantly predicted suicidality in the ASC group when controlling for the aforementioned variables. Hence, a clear recommendation for policy and practice to reduce suicide risk in autistic adults, a high-risk group for dying by suicide [15], is to urgently identify and address unmet support needs in this group. Meeting this shortfall in support could, at least in part, help reduce high rates of suicidality and death by suicide in the autistic community. Research from our group is exploring in more depth barriers and enablers in accessing treatment and support in autistic adults, to help assist in service planning.

The rate of NSSI in the ASC group (63.6%) was significantly higher than the general population group (29.8%), and similar to the rate reported in previous research [10] (50%), which also utilised the NSSI-AT in autistic adults. NSSI also significantly predicted suicidality in autistic adults, after controlling for a range of known risk factors. Hence, NSSI should not continue to be overlooked, or seen as part of ASC, and rather must be addressed in its own right. Our findings are therefore an important call to action for the research community and clinicians to increase understanding and support for those with ASC experiencing NSSI. However, future studies will need to explore whether this rate of NSSI in ASC adults remains stable, and explore the measurement properties of NSSI assessment tools in ASC.

The current study has a number of strengths as well as limitations. This study is the first to use measures of suicidality (SBQ-R) and NSSI (NSSI-AT) that have good evidence of validity, albeit not yet in autistic adults [7, 10]. There is a paucity of validated outcome measures for autistic adults, and using tools validated for the general population is an important stop gap until tools adapted for autistic people become available [7, 10, 51–53]. The current study was only cross-sectional, and it is unclear for example whether unmet support needs are a cause or consequence of suicidality. The current study focused on adults, without intellectual disability (ID), and it is unknown whether autism and autistic traits would similarly be a unique risk marker for those with co-occurring ID. Although autism, autistic traits, unmet support needs, and camouflaging explained significant additional variance in suicidality when statistically controlling for a number of other factors, the additional variance explained was small.

ASC diagnosis was assessed by self-report only; however, a majority of participants confirmed the clinic where this diagnosis was obtained. Lifetime suicide attempts in the general population (8%) and ASC group (38%) are similar to previous studies [2, 17], which suggests that the sample was not biased in this respect. However, lifetime experience of depression in the general population (44.9%) and ASC group (80%) were much higher than previous estimates [2, 22, 54], despite participants not being recruited because of experience with mental health problems. The rate of mental health difficulties in the current sample therefore may not be representative of the general or autistic populations. A majority of participants in the steering group and online survey were female. Therefore, it could be argued that the topics explored in the survey and study findings apply mostly to autistic females and may not be generalisable to autistic males. However, a majority of autistic males and autistic females reported camouflaging, and regression analyses statistically controlled for sex, suggesting this and other risk markers apply to both sexes.

A key strength and novel aspect of the current study was the participatory research element with a group of autistic adults, who refined the focus of the study, and the content of the survey. This ensured that the study included a range of possible unique and common risk factors for suicidality not explored or considered in previous research on this topic. It also ensured high content validity of the survey, which was refined through three iterations of feedback from the steering group. Previous research has shown that the views of the autistic community which the research affects are rarely included, which can hamper the potential benefits of ASC research for the wider community [11]. Our study demonstrates the importance of including the voices of autistic people in important and sensitive research that can impact their lives.

## Conclusions

The current study is the first to use validated assessment tools, and survey co-designed with autistic people, to explore unique risk factors for suicidality in this group. Results reiterate that rates of suicidality in autistic adults are higher than the general population, and ASC diagnosis and autistic traits are independent risk markers for suicidality. Importantly, unique risk markers for suicidality in ASC include camouflaging one’s ASC in order to fit in in social situations and number of unmet support needs. These explain small but significant additional variance in suicidality in ASC, above a range of known risk factors common with the general population. Future research must further explore these and identify other unique mechanisms driving suicidality in ASC to develop new effective suicide prevention strategies for this group.

## Abbreviations

ADHD: Attention deficit hyperactivity disorder
AQ: Autism-Spectrum Quotient
AS: Asperger syndrome
ASC: Autism spectrum condition
GP: General population
HFA: High functioning autism
ID: Intellectual disability
NSSI: Non-suicidal self-injury
NSSI-AT: Non-suicidal self-injury assessment tool
PDD: Pervasive developmental disorder
PDD-NOS: Pervasive Developmental Disorder Not Otherwise Specified
SBQ-R: Suicidal Behaviours Questionnaire-Revised

## References

[1] Zahid S, Upthegrove R (2017). Suicidality in autistic spectrum disorders: a systematic review. Crisis.

[2] Cassidy S, Bradley P, Robinson J, Allison C, McHugh M, Baron-Cohen S (2014). Suicidal ideation and suicide plans or attempts in adults with Asperger's syndrome attending a specialist diagnostic clinic: a clinical cohort study. Lancet Psychiatry.

[3] Segers M, Rawana J (2014). What do we know about suicidality in autism spectrum disorders? A systematic review. Autism Res.

[4] Hedley D, Uljarević M. Systematic review of suicide in autism spectrum disorder: current trends and implications. Curr Dev Disord Rep. 2018;5(1):65-76.

[5] Croen LA, Zerbo O, Qian Y, Massolo ML, Rich S, Sidney S, Kripke C (2015). The health status of adults on the autism spectrum. Autism.

[6] Cassidy S, Rodgers J (2017). Understanding and prevention of suicide in autism. Lancet Psychiatry.

[7] Cassidy S, Bradley L, Wigham S, Bowen E, Rodgers J. Measurement properties of tools used to assess suicidality in adults with and without autism spectrum conditions: a systematic review. Clin Psychol Rev. In Press

[8] Hannon G, Taylor EP. Suicidal behaviour in adolescents and young adults with ASD: Findings from a systematic review. Clinical psychology review. 2013;33(8):1197-204. 10.1016/j.cpr.2013.10.003 24201088

[9] Ribeiro JD, Franklin JC, Fox KR, Bentley KH, Kleiman EM, Chang BP, Nock MK (2016). Self-injurious thoughts and behaviors as risk factors for future suicide ideation, attempts, and death: a meta-analysis of longitudinal studies. Psychol Med.

[10] Maddox BB, Trubanova A, White SW (2017). Untended wounds: non-suicidal self-injury in adults with autism spectrum disorder. Autism.

[11] Pellicano L, Dinsmore A, Charman T (2013). A Future Made Together: Shaping autism research in the UK.

[12] Nock MK, Borges G, Bromet EJ, Alonso J, Angermeyer M, Beautrais A (2008). Cross-national prevalence and risk factors for suicidal ideation, plans and attempts. Br J Psychiatry.

[13] Kessler RC, Borges G, Walters EE (1999). Prevalence of and risk factors for lifetime suicide attempts in the National Comorbidity Survey. Arch Gen Psychiatry.

[14] O'Carroll PW (1992). Attempted suicide among young adults: progress toward a meaningful estimate of prevalence. Am J Psychiatr.

[15] Hirvikoski T, Mittendorfer-Rutz E, Boman M, Larsson H, Lichtenstein P, Bölte S (2016). Premature mortality in autism spectrum disorder. Br J Psychiatry.

[16] Upthegrove R, Abu-Akel A, Chisholm K, Lin A, Zahid S, Pelton M, Apperly I, Hansen PC, Wood SJ (2018). Autism and psychosis: clinical implications for depression and suicide. Schizophr Res.

[17] Pelton MK, Cassidy SA (2017). Are autistic traits associated with suicidality? A test of the interpersonal-psychological theory of suicide in a non-clinical young adult sample. Autism Res.

[18] Takara K, Kondo T (2014). Comorbid atypical autistic traits as a potential risk factor for suicide attempts among adult depressed patients: a case–control study. Ann General Psychiatry.

[19] Chen MH, Pan TL, Lan WH, Hsu JW, Huang KL, Su TP, Li CT, Lin WC, Wei HT, Chen TJ, Bai YM (2017). Risk of suicide attempts among adolescents and young adults with autism spectrum disorder: a Nationwide longitudinal follow-up study. J Clin Psychiatry.

[20] Barraclough BJ, Bunch J, Nelson B, Sainsbury P (1974). A hundred cases of suicide: clinical aspects. Br J Psychiatry.

[21] Wigham S, Barton S, Parr JR, Rodgers J (2017). A systematic review of the rates of depression in children and adults with high-functioning autism spectrum disorder. J Ment Health Res Intellect Disabil.

[22] Lever AG, Geurts HM (2016). Psychiatric co-occurring symptoms and disorders in young, middle-aged, and older adults with autism spectrum disorder. J Autism Dev Disord.

[23] Van Orden KA, Witte TK, Cukrowicz KC, Braithwaite SR, Selby EA, Joiner TE (2010). The interpersonal theory of suicide. Psychol Rev.

[24] Orsmond GI, Shattuck PT, Cooper BP, Sterzing PR, Anderson KA (2013). Social participation among young adults with an autism spectrum disorder. J Autism Dev Disord.

[25] Hedley D, Uljarević M, Wilmot M, Richdale A, Dissanayake C (2017). Brief report: social support, depression and suicidal ideation in adults with autism spectrum disorder. J Autism Dev Disord.

[26] Hedley D, Uljarević M, Wilmot M, Richdale A, Dissanayake C (2018). Understanding depression and thoughts of self-harm in autism: a potential mechanism involving loneliness. Res Autism Spectr Disord.

[27] Crane L, Chester JW, Goddard L, Henry LA, Hill E (2016). Experiences of autism diagnosis: a survey of over 1000 parents in the United Kingdom. Autism.

[28] Jones L, Goddard L, Hill EL, Henry LA, Crane L (2014). Experiences of receiving a diagnosis of autism spectrum disorder: a survey of adults in the United Kingdom. J Autism Dev Disord.

[29] Lai MC, Baron-Cohen S (2015). Identifying the lost generation of adults with autism spectrum conditions. Lancet Psychiatry.

[30] World Health Organisation. (2018). World Health Statistics. Global Health Observatory data. Available from: http://apps.who.int/iris/bitstream/handle/10665/272596/9789241565585-eng.pdf?ua=1. Accessed 21 Feb 2018.

[31] Lai MC, Lombardo MV, Ruigrok AN, Chakrabarti B, Auyeung B, Szatmari P, Happé F, Baron-Cohen S, MRC AIMS Consortium (2017). Quantifying and exploring camouflaging in men and women with autism. Autism.

[32] Hull L, Petrides KV, Allison C, Smith P, Baron-Cohen S, Lai MC, Mandy W (2017). “Putting on my best normal”: social camouflaging in adults with autism spectrum conditions. J Autism Dev Disord.

[33] Rynkiewicz A, Schuller B, Marchi E, Piana S, Camurri A, Lassalle A, Baron-Cohen S (2016). An investigation of the ‘female camouflage effect’in autism using a computerized ADOS-2 and a test of sex/gender differences. Mol Autism.

[34] Duerden EG, Oatley HK, Mak-Fan KM, McGrath PA, Taylor MJ, Szatmari P, Roberts SW (2012). Risk factors associated with self-injurious behaviors in children and adolescents with autism spectrum disorders. J Autism Dev Disord.

[35] South M, Ozonoff S, McMahon WM (2005). Repetitive behavior profiles in Asperger syndrome and high-functioning autism. J Autism Dev Disord.

[36] Whitlock J, Exner-Cortens D, Purington A (2014). Assessment of nonsuicidal self-injury: development and initial validation of the non-suicidal self-injury–assessment tool (NSSI-AT). Psychol Assess.

[37] Osman A, Bagge CL, Gutierrez PM, Konick LC, Kopper BA, Barrios FX (2001). The Suicidal Behaviors Questionnaire-Revised (SBQ-R): validation with clinical and nonclinical samples. Assessment.

[38] Baron-Cohen S, Wheelwright S, Skinner R, Martin J, Clubley E (2001). The autism-spectrum quotient (AQ): evidence from Asperger syndrome/high-functioning autism, males and females, scientists and mathematicians. J Autism Dev Disord.

[39] Ruzich E, Allison C, Smith P, Watson P, Auyeung B, Ring H, Baron-Cohen S (2015). Measuring autistic traits in the general population: a systematic review of the Autism-Spectrum Quotient (AQ) in a nonclinical population sample of 6,900 typical adult males and females. Mol Autism.

[40] Woodbury-Smith MR, Robinson J, Wheelwright S, Baron-Cohen S (2005). Screening adults for Asperger syndrome using the AQ: a preliminary study of its diagnostic validity in clinical practice. J Autism Dev Disord.

[41] Cohen J (1988). Statistical power analysis for the behavioral sciences.

[42] Field A. Discovering statistics using SPSS. London: Sage publications; 2009.

[43] Ratto AB, Kenworthy L, Yerys BE, Bascom J, Wieckowski AT, White SW, Wallace GL, Pugliese C, Schultz RT, Ollendick TH, Scarpa A. What about the girls? Sex-based differences in autistic traits and adaptive skills. J Autism Dev Disord. 2018;48(5):1698-711. 10.1007/s10803-017-3413-9 PMC5925757 29204929

[44] Gotham K, Bishop SL, Brunwasser S, Lord C (2014). Rumination and perceived impairment associated with depressive symptoms in a verbal adolescent–adult ASD sample. Autism Res.

[45] Heasman B, Gillespie A (2018). Perspective-taking is two-sided: misunderstandings between people with Asperger’s syndrome and their family members. Autism.

[46] Jaswal VK, Akhtar N (2018). Being vs. appearing socially uninterested: challenging assumptions about social motivation in autism. Behav Brain Sci.

[47] Sasson NJ, Faso DJ, Nugent J, Lovell S, Kennedy DP, Grossman RB (2017). Neurotypical peers are less willing to interact with those with autism based on thin slice judgments. Sci Rep.

[48] Milton DE (2012). On the ontological status of autism: the ‘double empathy problem’. Disabil Soc.

[49] Milton D, Sims T (2016). How is a sense of well-being and belonging constructed in the accounts of autistic adults?. Disabil Soc.

[50] Raja M (2014). Suicide risk in adults with Asperger’s syndrome. Lancet Psychiatry.

[51] Cassidy SA, Bradley L, Bowen E, Wigham S, Rodgers J (2018). Measurement properties of tools used to assess depression in adults with and without autism spectrum conditions: a systematic review. Autism Res.

[52] McConachie H, Parr JR, Glod M, Hanratty J, Livingstone N, Oono IP, Robalino S, Baird G, Beresford B, Charman T, Garland D. Systematic review of tools to measure outcomes for young children with autism spectrum disorder. https://ore.exeter.ac.uk/repository/handle/10871/17760. Accessed 21 Feb 2018. 10.3310/hta19410 PMC4781156 26065374

[53] Wigham S, McConachie H (2014). Systematic review of the properties of tools used to measure outcomes in anxiety intervention studies for children with autism spectrum disorders. PLoS One.

[54] Kessler C, Berglund P, Demler O, Jin R, Merikangas R, Walters E (2005). Lifetime prevalence and age-of-onset distributions of DSM-IV disorders in the national comorbidity survey replication. Arch Gen Psychiatry.

